# Credit and Recognition for Contributions to Data-Sharing Platforms Among Cohort Holders and Platform Developers in Europe: Interview Study

**DOI:** 10.2196/25983

**Published:** 2022-01-13

**Authors:** Thijs Devriendt, Pascal Borry, Mahsa Shabani

**Affiliations:** 1 Department of Public Health and Primary Care Faculty of Medicine KU Leuven Leuven Belgium; 2 Metamedica Faculty of Law and Criminology UGent Gent Belgium

**Keywords:** information dissemination, qualitative research, ethics, database management systems, cohort studies, science policy, incentives, rewards

## Abstract

**Background:**

The European Commission is funding projects that aim to establish data-sharing platforms. These platforms are envisioned to enhance and facilitate the international sharing of cohort data. Nevertheless, broad data sharing may be restricted by the lack of adequate recognition for those who share data.

**Objective:**

The aim of this study is to describe in depth the concerns about acquiring credit for data sharing within epidemiological research.

**Methods:**

A total of 17 participants linked to European Union–funded data-sharing platforms were recruited for a semistructured interview. Transcripts were analyzed using inductive content analysis.

**Results:**

Interviewees argued that data sharing within international projects could challenge authorship guidelines in multiple ways. Some respondents considered that the acquisition of credit for articles with extensive author lists could be problematic in some instances, such as for junior researchers. In addition, universities may be critical of researchers who share data more often than leading research. Some considered that the evaluation system undervalues data generators and specialists. Respondents generally looked favorably upon alternatives to the current evaluation system to potentially ameliorate these issues.

**Conclusions:**

The evaluation system might impede data sharing because it mainly focuses on first and last authorship and undervalues the contributor’s work. Further movement of crediting models toward contributorship could potentially address this issue. Appropriate crediting mechanisms that are better aligned with the way science ought to be conducted in the future need to be developed.

## Introduction

### Background

Data sharing in science maximizes the utility and impact of cohort data and, therefore, contributes to improving clinical practice and public health. In this paper, data sharing will be considered as “making data available to other researchers for carrying out scientific analyses.” This definition encompasses not only the transfer of data out of the institution but also the remote use of data from outside the institution (eg, through federated analyses). Data sharing also increases the accountability of researchers as others can rerun any analyses that are published or use different methodologies to answer the same research question to replicate findings [1]. Furthermore, data sets may be used to explore secondary hypotheses, for meta-analyses within systematic reviews, or for educational purposes [1]. Despite these advantages, academics from diverse fields have emphasized that broad data sharing is impeded by the lack of incentives for those who share data [2-5]. In this context, incentives for researchers are often understood as interventions that can stimulate researchers to engage in particular behavior—here, open science practices. They may compensate for the lack of recognition or rewards for those who share their data. Empirical studies on data sharing indicate that recognition and credit can be major concerns of researchers [2,6-9]. The salience of granting due credit was also underlined when the WorldWide Antimalarial Resistance Network data platform became operational. Within the WorldWide Antimalarial Resistance Network, many of those who generated data within their local contexts (hereafter referred to as *data generators*) only agreed to contribute after being offered coauthorship on papers for downstream data use [10].

Offering coauthorship has become a commonly used means of crediting data generators. Through this mechanism, the rising prevalence of multi-cohort, multicenter studies has also been driving authorship inflation over time and has contributed to the phenomenon of hyperauthorship [11,12]. The term hyperauthorship was coined in 2001 by Cronin [11], who used it to refer to articles with “massive coauthorship levels” [12]. In this paper, hyperauthorship will be considered as *>100 authors*. However, the validity of traditional crediting models has come under mounting pressure as it raises questions about credit misconduct, accountability of authors, and the undue influence of hyperauthorship on popular metrics of scientific productivity [11-13]. Multiple scholars have suggested that hyperauthorship has the double effect of multiplying the credit attributed to the production of knowledge and fragmenting the responsibility among all authors [13,14]. At the same time, the academic reputation of all the authors may still be affected by the errors or mistakes of 1 author, albeit the degree of impact depends on the author’s position [15]. A recent survey found that 46.6% and 37.9% of researchers reported having encountered questionable or unethical behavior with regards to author naming and ordering, respectively [16]. The highest degrees of dissatisfaction with author order, ghost authorship, and gift authorship are found among early-career academics and within health sciences [16,17].

In response to both challenges, namely academic recognition and accountability, alternative recognition approaches have been proposed, such as through designations that elucidate the contributions to scientific work in a more granular fashion [18,19]. At the end of the 1990s, Rennie et al [14] argued in favor of abandoning authorship and moving toward contributorship, where all contributions would be disclosed. Contributorship has the advantage of being able to more adequately recognize *specialist* authors (ie, those authors who contribute through 1 or 2 roles within research projects). In parallel, expert groups providing advice to policy makers are advocating moving away from commonly used and abused metrics, such as H-index and the journal impact factor, toward so-called *responsible* metrics [20]. The San Francisco Declaration on Research Assessment and the Leiden Manifesto for Research Metrics take similar positions on the development of novel indicators of scientific productivity and underscore the need to reconsider the relationship between quantitative (indicators) and qualitative (judgment) aspects of evaluation [21,22]. In recent years, these thoughts have started to gain traction and have become incorporated within policy documents on the open science agenda, such as documents of the working expert groups and mutual learning exercises of the European Commission [20,23-28]. Central to these documents is the understanding that incentives partly drive the behavior of scientists and that the installation of adequate incentives and rewards is essential to achieve open science practices (including greater data sharing).

### Objective

Although perspective pieces on these issues are plentiful, few qualitative studies have explored the views of scientists on potential modifications of the recognition system. Colledge et al [29] recorded the experiences of biobank stakeholders with publication credit in collaborative research. Pinel [30,31] described the dynamics of competition, credit, and financial resources in collaborations in epigenetics based on an ethnographic study. Sauerman et al [32] went further and conducted a survey on researchers’ responses on the perceived usefulness of contributorship statements for evaluation purposes, in which qualitative data on researcher perspectives were also collected. Empirical studies on data sharing and prior experiences with contributing to data-sharing platforms indicate that credit is an important factor. Therefore, our study explores the perspectives of cohort holders and platform developers on credit for data sharing and aims to describe concerns on this issue in detail within the context of cohort research. Furthermore, we asked researchers about their views on potential alterations to the reward system and gave them the opportunity to make suggestions for changes.

## Methods

### Overview

Qualitative methods were used to explore the views and opinions of cohort holders and platform developers. Cohort holders were understood to be those involved in generating the cohorts and platform developers to be those involved in designing data-sharing platforms. Most participants could be classified as cohort holders or both cohort holders and platform developers. They were recruited using a purposive sampling strategy that explored 3 European projects creating data-sharing platforms (euCanSHare [a European Union-Canada joint infrastructure for next-generation multistudy heart research], Common Infrastructure for National Cohorts in Europe, Canada, and Africa, and EUCAN-Connect). These 3 projects are funded under the same Horizon 2020 call. Contact persons for cohorts within the euCanSHare consortium were identified via a list of names acquired from the project manager and other lists found on the web. Within euCanSHare, nearly all European cohorts were contacted, except those for whom the principal investigator had passed away or where multiple cohorts were managed by the same center. Names of contact persons from the Common Infrastructure for National Cohorts in Europe, Canada, and Africa and EUCAN-Connect consortium were acquired by contacting the project coordinators or by querying the participating cohorts in databases and registering the first or last authors on cohort profiles or recent articles. Potential interviewees were contacted via email. Interviews were semistructured in nature and were conducted by TD using a semistructured interview guide. The interview guide was updated after conducting the first interview. Interviews were audio recorded, transcribed verbatim, deidentified, and analyzed using inductive content analysis in which content categories were derived from the data rather than being predetermined [33-35]. Transcripts were coded into narrow content categories using NVivo 12 software (QSR International). Subsequently, categories were compared, revised, and broadened through iterative exploration of the excerpts, the coding scheme, and the original transcripts. Upon completion, the coding scheme and extracts were checked by MS for consistency and rationale. The coding scheme was further revised upon receiving feedback. All participants signed written informed consent. The interviews were conducted on the web and recorded. In total, 17 interviews were conducted: of these, 13 (76%) with cohorts affiliated with euCanSHare and 4 (24%) with the other data-sharing platforms. The study was approved by the social and societal ethics committee at Katholieke Universiteit Leuven (G-2018 10 1348).

### Terminology

Cohort holders are those researchers who are apt at deciding on the use of cohort data in research projects.

Data generators are those researchers who have been involved in *any* aspect of the production of cohort data, such as conception, data collection, or quality assurance.

Data contributors are those researchers who contribute data to projects to be used by other scientists within the framework of multicohort, multicenter studies.

## Results

**Figure 1:**
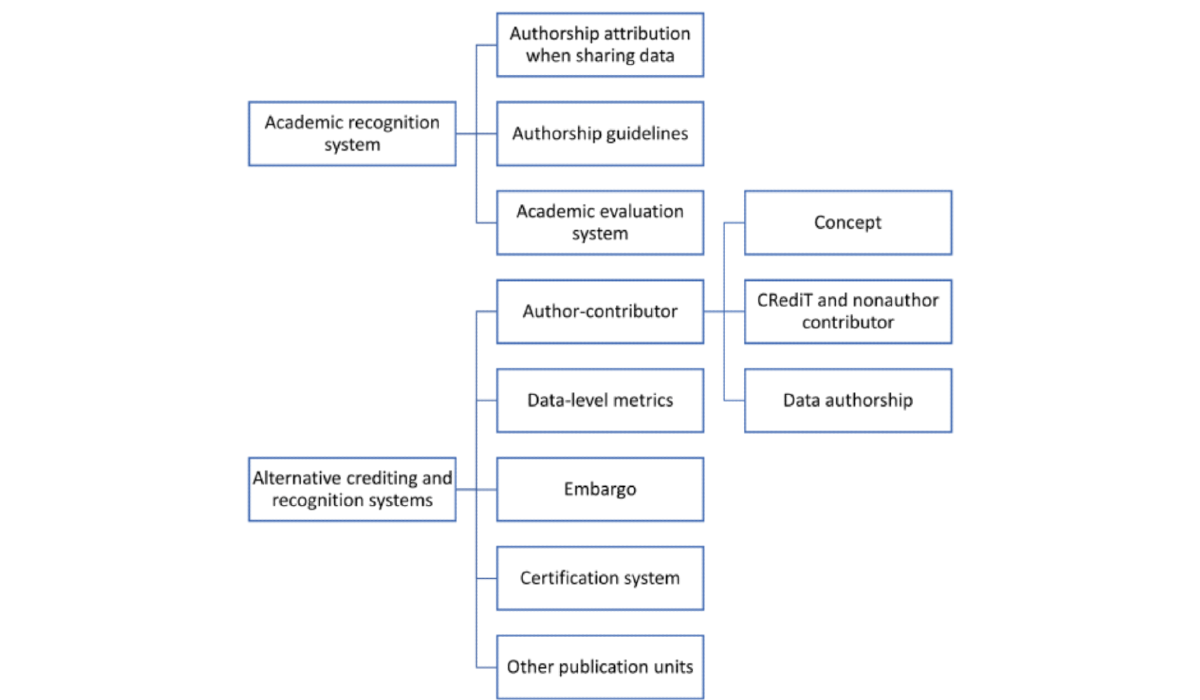
Coding scheme for the interview data. CRediT: contributor roles taxonomy.

The subsections of the interview transcripts pertaining to credit were classified into the following main categories: (1) authorship attribution when sharing data, (2) authorship guidelines and data sharing, (3) authorship and the academic evaluation system, and (4) alternative crediting and recognition mechanisms. The tree diagram for these categories is displayed in Figure 1.

### Authorship Attribution When Sharing Data

Most interviewees communicated that the sharing of data was usually rewarded with authorship. A minority of interviewees considered that they or persons within their research team did not need to be authors when sharing data but required formal citation (eg, through cohort profiles) or acknowledgment of the cohort. Most respondents stated that they only shared data when collaborating on downstream analyses and that the involvement of researchers affiliated with the study was required:

In general, [the researchers collecting] the cohorts are interested in sharing their data and participating in the analysis [...] They have created their cohorts for research and [...] they want to be part of the research and therefore authorship is important [...] In general, [researchers will not share if they do not get the opportunity to participate in the analysis]. It varies very much from center to center.

Interviewee 1

Interviewees explained that, within research consortia, various modes of working could be adopted. Within some consortia, all participating research teams are able to suggest proposals for analyses. First, the scientific validity of a research proposal would be reviewed by a central committee associated with the consortium. If it passed this stage, it would be subsequently distributed to all groups in the consortium whose data are to be used in the requested analysis. All these groups would then be offered the opportunity to collaborate in the research project and would have to explicitly confirm their participation. Several rounds of drafting and circulation of the data analysis plan among participants would follow, which allow active discussion of methodology and technical details. In contrast, within larger consortia, especially genetic research consortia, respondents argued that it might not be possible to involve all data contributors in the drafting of the data analysis plan. This created some frustrations among interviewees, as they would not be truly involved in the research:

Manuscript leaders should try to increase transparency over all over the work that they are doing because if I receive an advanced draft on a very complicated genetic issue with forty tables only in the supplementary material and maybe fifteen days to provide the feedback, this means: Please do not say anything, just check the affiliation [...] You cannot have a strict law or rule but it is a gentlemen’s agreement.

Interviewee 7

Respondents argued that often 1 or 2 persons per cohort would be allowed to become coauthors on resultant manuscripts. The need to limit the number of coauthors on these articles was mentioned by most respondents, and it was frequently referred to as a balance between the number of authors and the due credit to researchers for data production and sharing.

### Authorship Guidelines and Data Sharing

The interviewees indicated that receiving approval and comments from all coauthors was not straightforward. A minority of interviewees brought up that, within consortia, the discussion had arisen about whether researchers who have never responded to emails requesting commentary should still be listed as coauthors:

You send them the manuscript with the complete analysis, written and ready to be submitted, asking them to read and make comments. There are people that in five, six years, have never answered. Not just to comment but to say: “Okay, I received the manuscript and this is okay for me.”

Interviewee 13

Of the 17 respondents, 1 (6%) respondent argued that for older cohorts, the researchers involved in the original data collection might not be available anymore, for example, because of retirement. This means that persons managing that cohort might not have been involved in the acquisition of the original data. This makes it more complicated for researchers to conform with the first criterion of the International Committee for Medical Journal Editors (ICMJE) guidelines, which stipulates that authors need to have delivered “substantial contributions to the conception or design of the work; or the acquisition, analysis, or interpretation of data for the work” [36]. Nevertheless, interviewees described experiences where cohort holders simply requested coauthorship regardless of compliance with the authorship guidelines.

Of the 17 respondents, 1 (6%) respondent pointed out that limiting the number of coauthors per cohort might mean that some researchers who have contributed are not recognized. At the same time, if a center contributes multiple cohorts, more names might be put on research articles than those that would qualify for authorship:

It is also maybe a coincidence whose name will be on this list. For example, if we have five cohorts in a collaboration, then we can put ten names on the article and the first two or three will be someone that will actually have something to do with the data but the last five or seven names will be like: pick one.

Interviewee 11

Respondents generally considered that for multi-authored papers, the authors who contributed data could only be accountable for their contributions rather than for the whole content of the paper or papers. This means that authors must be able to vouch for the integrity of the data and the proper conduct of any analyses that were performed locally. They can also review the manuscript for overinterpretations, errors in the background information, and an accurate description of the cohort itself. Respondents mentioned that, apart from their own contributions, one should simply put trust in others. Notably, a respondent referred to the need to have data contributors as coauthors, as the group leading the analysis cannot take responsibility for the proper handling of the data if they do not understand the particularities of the data.

Interviewees considered that data contributors might not be able to exert sufficient influence to correct potential errors in the manuscript or request additional analyses. Mistakes in the reporting of the cohort might reveal that data were incorrectly handled; however, redoing the analysis and reworking the tables could be very time consuming and substantially delay publication. Researchers might be disincentivized to comment if they anticipate that their views will not be duly considered or if they feel that others might blame them for causing time delays. Those leading the analysis might give little weight to comments if these comments are not shared by other researchers.

### Authorship and the Academic Evaluation System

The aforementioned difficulties with claiming authorship and responsibility might create problems for acquiring recognition: if authorship is considered unjustified or too easily obtained, it loses its value. Most respondents argued that, for evaluation purposes, the first and last authors are considered to be most important, with little focus on other positions. Although some respondents stated that they were not considered for evaluation purposes at all, others expressed confidence in the first, last, and middle authorship being appropriately weighted. Of the 17 interviewees, 3 (18%) reported experiences with funders or evaluation panels being critical of a large number of papers with extensive author lists and that these funders and evaluations panels would not recognize the value in such authorship when no specific *evidential* contributions were reported:

When I tried to become a research director, it was good that I valorized the data and the fact that I had many publications thanks to this, contributed to me to go through the first step so my file was selected [and] I could defend my project in front of the jury. But once I was [there] the [fact] that I was mainly sharing data and not conducting my own scientific projects was a very bad thing. [...] I had difficulties defending [these actions] because it seemed that I was just a platform and not really directing some important research.

Interviewee 16

Interviewees raised concerns over the lack of recognition for data generators and researchers with specialized roles within our current system. According to these interviewees, the recognition system in academia is geared primarily toward clinical researchers and less toward other types of researchers such as statisticians and data generators:

Researchers who contribute through data sharing or the creation of data that then others use, I think that needs to be acknowledged and is not really acknowledged in the middle of a paper. [...] There is a need to be able to recognize people who are doing more of the hands-on work, the less research-type work, they are building the resource. That is not recognized to the same extent as then using the data and scientific output. [...] [I would prefer] if funders were more willing to fund support persons rather than just research, post-docs and clinical [persons] who all have the incentive to publish in the first and last authorship role.

Interviewee 12

Respondents argued that junior researchers are disadvantaged by this system, as they are in need of publications in which they are the first author. They are often not experienced enough to lead the analysis of a large number of cohorts, and when collaborating, they would only be in positions where their work remains largely unrecognized. In addition, junior researchers might intend to perform a similar analysis on local cohort data as planned by the consortium, which would threaten their publication opportunities. At the same time, it is often the junior researchers who might have dedicated a substantial part of their time to collecting data or performing quality assurance.

Despite the difficulties surrounding authorship in terms of attribution, accountability, and credit, most interviewees generally stated that they were satisfied with the ICMJE or Vancouver guidelines and considered that they form an appropriate basis for discussing authorship disputes. Many respondents held pragmatic views on offering authorship, which, they argued, recognizes scientific work or stimulates data sharing to the benefit of science. Among the interviewees who suggested changes, two opposing views could be identified: (1) authorship should be more inclusive of data generators and those in supporting roles and (2) authorship should be only for those who have actively and intellectually contributed to the analysis. Adherents to the former view based their argumentation on the inequity of unrecognized work by specialists or data generators. Adherents to the latter view based their argumentation primarily on authorship losing its value or going against what it used to be.

### Alternative Crediting and Recognition Mechanisms

#### Contributor Roles Taxonomy and Data Authorship

Researchers were asked to state their opinions on multiple proposals to reform the academic recognition system. Most respondents declared their willingness to explore alternatives to authorship, such as the specification of contributorship roles. Respondents considered that this could resolve the concerns around accountability to display that one has contributed substantially to articles with extensive author lists and allow better recognition of specialized researchers, such as those contributing to only 1 or 2 tasks within large collaborations. The principal downsides perceived by participants were related to increased bureaucracy and complexity for evaluation purposes. Some argued that contributor roles taxonomy (CRediT) could disincentivize credit misconduct by forcing authors to declare their contributions, whereas others argued that it does not make a difference, as boxes may be very easily ticked without subsequent verification. Of the 17 respondents, 1 (6%) respondent stated that when data were generated, the name of the data generator was added to the code. This concept of *data authorship* can then make persons eligible for authorship when the said data are reused. Although respondents were generally open to alternatives, many explained that their views would depend on the manner in which these systems are eventually used for evaluation purposes:

[CRediT] is an interesting concept. It has some potential. On the other hand, I would not like to see things getting to complicated, too long lists, different alternatives. There are then differences in how people in different countries interpret those boxes [with different roles]. It might be good but it should be kept relatively short and relatively clear.

Interviewee 2

Nevertheless, some respondents considered that the lack of recognition of these categories by universities and funders could create barriers to contributing to research. Researchers might be dissuaded from engaging in activities that do not provide credit:

The introduction of non-author contributors was a very big step forward. Now the problem is within the scientific community, some universities whose actions are telling [you] that your work is recognized only if you are an author. They create barriers for the researchers to contribute to research.

Interviewee 1

#### Certification Systems, Data-Level Metrics, and Alternative Publication Units

In addition to these designations related to publications, interviewees suggested several systems to confer greater credit upon those who generate valuable data. Of the 17 respondents, 1 (6%) respondent argued that recognition systems could be set up by funders or institutions to recognize the broader merit of cohort studies, such as the way in which it actively involves participants, the quality of the data collection, and the way in which it disseminates relevant expertise. Such a certification system could include evaluating study practices according to internationally recognized criteria.

Approximately 18% (3/17) of participants argued in favor of the implementation of data-level metrics related primarily to either data quality or data use. Of the 17 interviewees, 1 (6%) interviewee argued that funders should shift toward indicators of high-quality data earlier in the process of data generation, which compares data with a fixed set of quality indicators. Other interviewees considered such metrics to be highly context dependent, not always useful for evaluation purposes, or to potentially instigate undesirable modes of competition (eg, by stimulating untruthful behavior). Some respondents considered that data use metrics could be relevant for decision-making. For example, they could be used to create *data set profiles* displaying collaborative sharing, data contribution, and sharing for educational and training purposes or partnerships with institutes in other countries.

Of the 17 participants, 1 (6%) participant argued in favor of publishing other publication units, such as the data dictionary and study protocol, in dedicated journals. Data generators are then to be evaluated based on the outputs that are more directly related to their work.

## Discussion

### Principal Findings

Our study indicates that granting authorship is commonly used to reward those who produce and share data. However, there is substantial divergence in the degree of involvement, from being actively involved in drafting data analysis plans or performing local analyses to sharing data without subsequent involvement in downstream analysis. When sharing data, the number of authors per cohort may be restricted to keep author lists more concise. Multiple challenges can arise with regards to the application of the ICMJE guidelines, such as when collaborators do not respond to requests for revision of manuscripts or when researchers who collected the data have already retired. It can be difficult to acquire credit for papers with extensive author lists if evaluation procedures for tenure or funding acquisition put excessive focus on first or last authorship. Those scientists in roles that are more prone to end up in middle author positions may be disadvantaged. Furthermore, researchers may be, in some instances, criticized for spending too much time on sharing data rather than leading the research themselves. Researchers look upon alternative crediting systems as potential solutions for addressing concerns over accountability or credit but refrain from supporting these endeavors wholeheartedly when the details of their implementation and its consequences remain unclear.

### Comparison With Previous Work

To some extent, academic credit influences researchers’ decisions to share data and the manner in which the sharing takes place. As interviewees indicated, there is an opportunity cost associated with spending time on data sharing, as this time could be spent on leading research projects instead. If data sharing is supported by a dedicated staff (eg, for data harmonization) or if administrative procedures are centralized, this factor would become less important. The choice between the 2 activities will then be partly influenced by the perception of rewards for data sharing in comparison with data analysis. If evaluation systems, formally through metrics or informally through qualitative evaluation, are tailored to primarily evaluate data analysis, the use of (local) resources could be considered more rewarding than contributing to collaborative work. Tornetta et al [37] described this dilemma between publishing smaller, less clinically relevant studies based on local data or contributing to large research groups and potentially jeopardizing one’s career. In contrast, Pinel [30,31] emphasizes the collaborative imperative in contemporary medical science but argues that engaging in collaborative work (eg, by joining consortia) is accompanied by the monopolization of that data and forced exclusion of outsiders. In addition to gaining credit for data sharing, this suggests that data sharing may still serve as the buy-in mechanism of acquiring access to high-quality data of others (either through reciprocal data sharing one on one or in consortia).

Multiple scholars have voiced concerns over recognition systems that may disincentivize scientists from contributing to multicenter, multidisciplinary research [13,37-39]. A recent analysis of guidelines for promotion within biomedical faculties revealed that few universities mention middle authorship or data sharing as valuable criteria [40]. Within biomedical sciences, special significance is often attached to first and last authorship, with the exception of large collaborations where more focus is on the ones in the beginning (eg, first and second) and the end of the authorship list (last and second-to-last) [41]. However, Mongeon et al [42] found that, for biomedical sciences and clinical medicine, the fraction of middle authors in an article is not connected to the total number of coauthors, implying that authorship inflation is not only because of an increase in the number of specialized contributions (eg, those who only contribute data). At the same time, clinical research and biomedical sciences possess a great degree of division of labor, with many researchers contributing to only 1 or 2 specific tasks [13,43]. These results suggest that focusing exclusively on first or last authorship for evaluation purposes could be detrimental to those authors who contributed to many tasks but do not take the first or last position and those who make many specialized contributions to various articles. Mazumdar et al [44] argued that the focus on first authorship is suitable for evaluating the work of small research groups, yet that this can be a disadvantage to typical team scientists, such as biostatisticians. Researchers may consider that certain types of contributions tend to be undervalued, such as technical analysis, model creation, and statistical analysis [45]. Through ethnographic studies, Pinel [30,31] describes the process in (epigenetic) cohort research, where PhD candidates and research nurses (and to some extent, postdoc holders) are involved in the *invisible* processes of data production, data curation, and *housekeeping jobs* of the laboratory. Pinel et al [46] criticize that engaging in these tasks has low prestige and is perceived as taking away from the *real science* and writing of publications. As suggested during the interviews, last authorship is considered particularly important for tenure as it emphasizes the independence of the researcher. Soares [47] has criticized the practice of attributing too much weight to independence as a criterion and misrecognizing the existing interdependence between researchers within collaborative work. The fact that many proposals for alternative crediting systems capitalize upon the perceived lack of recognition for specialized contributors merely underscores the salience of these issues [48].

One such crediting system that has substantially influenced authorship and gained popularity is contributorship. In the late 1990s, Rennie et al [14] proposed abandoning authorship and adopting contributorship in response to (1) changes in authorship practices and the way scientific research is conducted, (2) concerns about the dilution of accountability of authors, and (3) as an attempt to recognize all the authors who contributed more equitably. Contributors would simply be those who have contributed substantially and agree to take responsibility for the contribution without having to be involved in writing or revising the manuscript. In addition, guarantorship would be introduced, which refers to those who have contributed substantially to the article and have made efforts to organize, oversee, and double-check the integrity of the work [14]. Although its adoption was initially limited, it has become increasingly common in journals in the field of biomedicine over time. In 2015, the CRediT was introduced to standardize contributorship statements [49]. The use of CRediT has become more common in recent years and is anticipated to increase substantially in the near future [50]. The use of CRediT is argued to have advantages such as enabling meta-research programs, reducing credit misconduct, facilitating cross-disciplinary collaboration, and appropriately recognizing specialist roles and the contribution of software or data [18]. Within this interview study, researchers looked positively upon contributorship to elucidate who has contributed what to the research article, facilitate acquiring credit for specialists, and designate accountability. Along the same lines, Sauermann and Haeussler [32] found in their survey that >90% of researchers consider that contributorship statements add at least some additional information, with 40% seeing considerable or much additional information. They also found that 45% of respondents favor authorship order for the evaluation of researchers, whereas 18% would give greater weight to contributorship statements, with junior scientists attributing comparatively more weight to contributorship statements than senior scientists [32]. The lack of necessity, increased bureaucracy, divergent interpretation of roles across groups, lack of universal adoption, difficulty in accessing aggregate data, and the inability to divide teamwork were touched upon at least once during the interviews. The supplementary data of the survey by Sauermann and Haeussler [32] revealed similar concerns of scientists, with the addition of lack of detail (eg, weight or categories), risk of introducing conflict, and lack of experience in using alternative schemes for evaluation. Despite these potential drawbacks, contributorship could still evolve over time. For example, some scholars argued in favor of contributorship statements indicating both absolute (ie, form) and relative contributions (ie, weightage) [32,41,51]. CRediT could also evolve as science progresses or could be tailored to become discipline specific [50]. McNutt et al [52] recommended that authorship roles be embedded within author metadata to allow indexing and abstracting, which would facilitate their use for evaluation purposes.

The opposing views put forward by interviewees that authorship should (1) reaffirm its more *traditional* meaning by encompassing only those who have contributed intellectually to the analysis or (2) be expanded to include data generators and those in supporting roles should be understood from the history and evolution of data sharing within the ICMJE guidelines. Before the 2000s, the first criterion of the ICMJE guidelines stated that acquiring authorship required “substantial contributions to (1) conception and design, or analysis and interpretation of data” whereas explicitly stating that the collection of data itself did not justify authorship [53]*.* In May 2000, the guidelines were revised, and the acquisition of data was added to the first criterion [54]. In the version of August 2013, the statement that the collection of data itself does not merit authorship was omitted [55]. Finally, in 2017, the guidelines stipulated that data generation and sharing deserve substantial credit; data generators must be given the opportunity to collaborate; and if this is impossible, unpractical, or undesirable, the efforts of those who shared data should still be recognized [56]. A comparable reversal can be observed in the notion of accountability. Before the 2000s, all authors were responsible for the entirety of the work [53]*.* In May 2000, authors became responsible for “appropriate portions of the text” [54]. As of August 2013, authors are accountable for the parts of the work that they had performed and for which they needed confidence in the contributions of others [55]. Thus, the conception of authorship-worthy contributions has broadened to encompass data sharing, whereas the notion of accountability has narrowed to resolve the problems created by far-reaching specializations in science. In this sense, authorship has edged closer to contributorship over the past 20 years. With this understanding, the normative positions taken by interviewees on authorship simply reflect support for or opposition to this shift. It remains uncertain what the precise connection will be between authorship and contributorship in the future. As Smith and Master [43] have argued, perhaps contributorship will not replace authorship but will become the basis for it.

Finally, any reformation of the academic evaluation system should consider how research pipelines will commonly be organized in the future. A true shift from local to big data epidemiology could entail fundamental policy changes, such as more emphasis on centrally driven initiatives, fewer population studies being supported, or dedicated funding streams for data analysis [57,58]. Therefore, commentators have raised questions about how the academic interests of original investigators and data analysts will be balanced in the future and how participation in multicenter, international epidemiological studies would be seen in terms of career advancement [57,58]. Some have expressed concerns that the shift toward big data epidemiology will preclude junior researchers from opportunities in first-author articles or that credit and resources will be accumulated (ie, the Matthew effect) in fewer hands [59,60]. Contributions to data-sharing platforms are not disconnected from this social reality of academia, where academic competition and the acquisition of credit partly influence researchers’ behavior. Therefore, it remains essential that robust science policies, including sound recognition systems, can support these platforms to ensure that their full potential is realized.

### Limitations

Many of the recruited interviewees actively participated in collaborative research within the existing consortia. Therefore, positive experiences with such modes of working may have steered the opinions in favor of active collaboration. With cohorts outside of euCanSHare, interviewees held more heterogeneous views, especially when data generation was funded for the principal purpose of sharing data broadly, when already having experiences with progressive evaluation criteria, or when faced with higher degrees of centralization of research governance. As such, the discussion on incentives for data sharing is likely to be *highly* context dependent, with cultural differences existing even within different branches of cohort research and researchers being subject to different national, institutional, or departmental regulations and rules. This interview study probed the views of researchers on novel crediting systems while providing a minimal explanation of recent evolutions in terms of authorship, contributorship, other crediting systems, or their place within the open science agenda. Therefore, engaging with researchers in deliberation, where the flaws of science policy, the history of particular initiatives, the strengths and shortcomings, and policy evolutions in open science are explained, may still shift opinions.

### Conclusions

The acquisition of credit influences the degree and mode of sharing. The evaluation system might impede data sharing through the undervaluation of scientific work as contributors. The challenges posed by an increase in collaborative science led to the suggestion of adopting contributorship. Subsequently, the authorship model has changed positions on accountability and credit for data production and sharing, whereas contributorship has become standardized and increasingly common. These evolutions underscore the salience of providing due credit to data generators and team scientists. Contributorship statements may be able to better support recognition systems for data-intensive and collaborative science. Contributorship may further evolve by becoming discipline specific, incorporating relative weight or allowing indexation. Researchers might accept further movement toward contributorship, although the final test will be its utility for evaluation purposes.

## Abbreviations

CRediT: contributor roles taxonomy
ICMJE: International Committee for Medical Journal Editors
